# Bioinformatics-based design of a fusion vaccine with CTLA-4 variable region to combat Brucella

**DOI:** 10.1590/1414-431X2023e12938

**Published:** 2023-07-21

**Authors:** W.H. Guo, Y.J. Zhu, G. Haimiti, X.R. Xie, C. Niu, M. Li, J. Shi, Z.W. Yin, M.K. Yu, J.B. Ding, F.B. Zhang

**Affiliations:** 1 The First Affiliated Hospital of Xinjiang Medical University, Urumqi, Xinjiang, China; 2 Department of Reproductive Assistance, Center for Reproductive Medicine, The First Affiliated Hospital of Xinjiang Medical University, Urumqi, Xinjiang, China; 3 Department of Clinical Laboratory, The First Affiliated Hospital of Xinjiang Medical University, Urumqi, Xinjiang, China; 4 State Key Laboratory of Pathogenesis, Prevention, and Treatment of Central Asian High Incidence Diseases, The First Affiliated Hospital of Xinjiang Medical University, Urumqi, China; 5 School of Life Science and Technology, Southeast University, Nanjing, China

**Keywords:** CTLA-4 extracellular domain, Brucella, Bioinformatics approaches, Fusion vaccine

## Abstract

Brucellosis has become a global zoonotic disease, seriously endangering the health of people all over the world. Vaccination is an effective strategy for protection against *Brucella* infection in livestock in developed countries. However, current vaccines are pathogenic to humans and pregnant animals, which limits their use. Therefore, it is very important to improve the safety and immune protection of *Brucella* vaccine. In this study, different bioinformatics approaches were carried out to predict the physicochemical properties, T/B epitope, and tertiary structure of Omp2b and Omp31. Then, these two proteins were sequentially linked, and the Cytotoxic T lymphocyte associated antigen-4 (CTLA-4) variable region was fused to the N-terminal of the epitope sequence. In addition, molecular docking was performed to show that the structure of the fusion protein vaccine had strong affinity with B7 (B7-1, B7-2). This study showed that the designed vaccine containing CTLA-4 had high potency against *Brucella*, which could provide a reference for the future development of efficient brucellosis vaccines.

## Introduction

Brucellosis is a zoonotic infectious disease caused by *Brucella*, which mainly infects livestock (1). There are more than 500,000 new cases of brucellosis worldwide every year (2). Since 2000, brucellosis infection in China has rapidly spread from northern livestock areas to coastal and southeastern areas, showing a continuous upward trend (3). Symptoms of Brucella infection tend to be non-specific and include flu-like symptoms such as headache, joint pain, night sweats, fatigue, and anorexia, which is easily misdiagnosed as the common cold. If not treated in time, the course of disease can last several years, mainly manifested as arthritis, epididymal orchitis, neuro spondylitis, liver and spleen abscesses, and endocarditis; severe cases may die (4).

*Brucella* is a Gram-negative facultative intracellular parasite, which can be divided into many different species depending on host preference. Clinically, *B. melitensis*, *B. abortus*, and *B. suis* are of the greatest significance (5).

In view of the serious losses caused by brucellosis, many countries and regions in the world have formulated specific prevention, control, and eradication plans (6). In China, vaccination and eradication are the principles of comprehensive measures to prevent and control brucellosis. At present, the vaccines used for human brucellosis immunization mainly include 19-BA vaccine and 104M vaccine. The live attenuated vaccines used for animal brucellosis prevention and control include SL9, Rev. 1, RB5l, S2, and M5 (7). Although the current brucellosis vaccine plays a role in controlling the epidemic of brucellosis, there are a number of shortcomings with regard to short protection time, the serological test cannot distinguish immunity/infection, and virulence recovery (8). Therefore, a safe, stable, and efficient fusion vaccine must be designed. The screening, improvement, and verification of its effect have become the focus of brucellosis vaccine research.

Outer membrane proteins play an important role in bacterial differentiation, energy signal transmission, and material transport. They are the main components of biofilm function (9). Currently, some subunit proteins of *Brucella*, especially outer membrane proteins (Omps), such as Omp2b, Omp31, are candidates for vaccines (10,11). However, previous studies showed that vaccines constructed from a single protein stimulate poor immune responses and that multiple protein combinations enhance the vaccine's immune response. Therefore, the combination of Omp2b and Omp31 could be a good candidate to enhance the immune response against *Brucella*.

Cytotoxic T lymphocyte associated antigen-4 (CTLA-4) plays an important regulatory role in T-cell activation (12). Both B7-1 (CD80) and B7-2 (CD86) bind to CTLA4, which shares 30% homology with CD28 but has a 10 to 20 times greater binding affinity to the B7 molecules. CTLA-4 variable region (IgV_CTLA-4) can efficiently bind to B7 molecules on antigen-presenting cells (APCs), which can improve the affinity with APCs (13). Therefore, vaccines containing Omp2b, Omp31, and IgV_CTLA-4 can elicit good immune response by increasing antigen-presenting ability of APCs. In this study, a bioinformatics approach was used to predict the physical, chemical properties, structure, and the B-cell and T-cell antigenic epitopes of Omp2b and Omp31. A linker was used to connect the IgV_CTLA-4 with antigen epitopes to construct a fusion vaccine. Eventually, molecular docking and statistical analysis methods were used to assess the interaction between the fusion protein vaccine and human B7-1 and B7-2.

## Material and Methods

### Amino acid sequences of proteins

The GenBank online database was used to obtain the amino acid sequences of Omp2b, Omp31, and CTLA-4. GenBank is the NIH genetic sequence database (USA), an annotated collection of all publicly available DNA sequences [https://www.ncbi.nlm.nih.gov/genbank/] (14).

### Physicochemical parameters of proteins

The online software ProtParam (web.expasy.org/protparam/) was used to analyze the physicochemical parameters of Omp2b and Omp31 proteins, including atomic composition, molecular weight, theoretical isoelectric point (pI), polar charge, stability, hydrophobicity (the overall average value of the GRAVY range is between -2 and 2, and negative values represent hydrophilic proteins).

### Prediction of signal peptide sequence of proteins

When the signal peptide sequence is synthesized, it is recognized by the signal recognition particle (SRP), and the protein synthesis is suspended or slowed down. The signal recognition particle carries the ribosome to the endoplasmic reticulum, and the protein synthesis starts again. Under the guidance of signal peptide, the newly synthesized protein enters the endoplasmic reticulum cavity, and the signal peptide sequence is removed under the action of signal peptidase. Therefore, it is necessary to exclude the signal peptide sequence when predicting T/B epitopes.

The online server signalP-5.0 (SignalP-5.0 - DTU Health Tech, Denmark) was used to analyze the signal peptide of proteins Omp2b and Omp31.

### Prediction of transmembrane domains

The online server TMHMM (www.cbs.dtu.dk/services/TMHMM-2.0/), which has been rated best in an independent comparison of programs for prediction of TM helices (15), was used to analyze the transmembrane domains of proteins Omp2b and Omp31.

### Analysis of T-cell epitope of proteins

The vaccine exerts its immune effect mainly by producing antibodies, and the production of antibodies is mainly done by CD4+ T-cell cells. Therefore, only CD4+ T-cell epitopes need to be predicted. The MHC was called human leukocyte antigen (HLA) in humans, the HLA-DRB1*0701 (16.35%), HLA-DRB1*1501 (8.65%), and HLA-DRB1*0301 (7.69%) are the high frequency HLA alleles in Xinjiang, China (16), thus we selected the allele (the HLA-DRB1*0701) with the highest frequency as the prediction material, after removing the signal peptide sequence. The online software IEDB-MHCII (tools.iedb.org/mhcii/) was used for analyzing the T-cell epitopes of Omp2b and Omp31.

### Analysis of B-cell epitope of proteins

After removing the signal peptide sequence, the antigen dominant epitopes of B-cells of Omp2b and Omp31 were analyzed by IEDB (http://tools.iedb.org/bcell/). Epitopes with length of less than 5 units were excluded.

### Tertiary structure prediction of the Omp2b and Omp31 proteins

The homology of all protein models found was less than 30% in the Omp2b and Omp31 proteins, and their amino acid length is more than 200 aa. We chose the online server Robetta (https://robetta.bakerlab.org) to establish a model to analyze the tertiary structure of proteins Omp2b and Omp31. The online server first identifies structural templates from the PDB by the multiple threading approach LOMETS, with full-length atomic models constructed by iterative template-based fragment assembly simulations. Function insights of the target are then derived by re-threading the 3D models through protein function database BioLiP (17). Then, the online server SAVES v6.0 (https://saves.mbi.ucla.edu/) was used to evaluate the rationality of the predicted protein. In addition, we used the Discovery Studio (DS) software (a new molecular modeling environment for personal computer and professional molecular simulation software in life science) to edit its tertiary structure and mark its T/B epitope.

### Analysis of epitope characteristics of the Omp2b-Omp31 fusion protein

#### Amino acid sequence of the Omp2b-Omp31 fusion protein

The connection between Omp2b and Omp31 usually needs the help of connecting peptides to avoid mutual interference and affect the stability of the overall structure and function (18). Due to the stability of the secondary structure and the non-extensibility and bending characteristics, connecting peptides are often used to connect the distance between the two ends of the functional protein to ensure the integrity of the functional domain. At the same time, due to the controllable distance, the fusion protein can be adjusted to optimal activity and stability (19). Linker peptide refers to a segment of peptide existing between modules. Its length ranges from several to hundreds of amino acid residues (generally 5∼25). Common linker peptides can be divided into two categories: flexible and rigid (20). The most commonly used flexible linker peptide in fusion protein technology is the (GGGGS) n sequence proposed by Huston et al. (21). Therefore, Omp2b and Omp31 were connected by the linker (GGGGS)3 one by one.

#### Analysis of T-cell epitope of the Omp2b-Omp31 fusion protein

The method is the same as above. We used the online software IEDB-MHCII to analyze the T-cell epitopes. Finally, the T-cell epitopes of the fusion protein were compared with those of Omp2b and Omp31. We observed whether there were differences among them.

#### Analysis of B-cell epitope of the Omp2b-Omp31 fusion protein

The method is the same as above. We used the online software IEDB to analyze the B-cell epitopes. Finally, the B-cell epitopes of the fusion protein were compared with those of Omp2b and Omp31. We observed whether there were differences among them.

#### Tertiary structure prediction of the Omp2b-Omp31 fusion protein

Similarly, we established a model to analyze the tertiary structure of the protein Omp2b-Omp31. In addition, we used the DS software to edit its tertiary structure and mark its T/B epitope.

### Analysis of epitope characteristics of the IgV_CTLA-4-Omp2b-Omp31 fusion protein

#### Amino acid sequence of the IgV_CTLA-4-Omp2b-Omp31 fusion protein

CTLA-4 is involved in the transmission of immune signals as a transmembrane receptor on T lymphocytes. CTLA-4 and ligand B7 on the surface of APCs are a pair of synergistic costimulatory signals, which play a regulatory role in the activation of T-cells (22). Based on previous research, we used Pet30a (+) skeleton structure to simulate the linker sequence. Between CTLA-4 and protein Omp2b-Omp31, there are 16 amino acids (GTDDDDKAMADIGSEF), which is part of Pet30a (+). In this way, we ensured that the two proteins before and after fusion could fold correctly against the interference of steric hindrance.

#### Analysis of T-cell epitope of the IgV_CTLA-4-Omp2b-Omp31 fusion protein

We used the online software IEDB-MHCII to analyze the T-cell epitopes. Finally, the T-cell epitopes of the fusion protein were compared with those of Omp2b, Omp31, and Omp2b-Omp31, and differences among them were recorded.

#### Analysis of B-cell epitope of the IgV_CTLA-4-Omp2b-Omp31 fusion protein

We used the online software IEDB to analyze the T-cell epitopes. Finally, the B-cell epitopes of the fusion protein were compared with those of Omp2b, Omp31, and Omp2b-Omp31, and differences among them were recorded.

#### Tertiary structure prediction of the IgV_CTLA-4-Omp2b-Omp31 fusion protein

In the same way, we established a model to analyze the tertiary structure of the protein IgV_CTLA-4-Omp2b-Omp31. In addition, we used the DS software to edit its tertiary structure and mark its T/B epitope.

### Molecular docking

In order to analyze the interaction between *Brucella* fusion protein vaccine and human B7-1 and B7-2, 3D models of B7-1 (PDB ID 1DR9) and B7-2 (PDB ID 1NCN) were searched using the Protein Data Bank (PDB) (https://www.rcsb.org/). Then, we used the DS software to isolate the compounds and obtain the final mode since most of the results were compounds.

We used the ClusPro server (https://cluspro.org), a widely used tool for protein-protein docking, to dock Omp2b-Omp31 and IgV_CTLA-4-Omp2b-Omp31 with B7-1 and B7-2 molecules. The advantage of the software is that it can offer a number of advanced options to modify the search that include the removal of unstructured protein regions, applying attraction or repulsion, accounting for pairwise distance restraints, considering small angle X-ray scattering (SAXS) data, and finding heparin binding sites (23).

Finally, the results (the lower the score, the stronger the affinity) were statistically analyzed by GraphPad Prism 9 software (USA) to assess differences in scores and to determine whether the affinity was stronger with the addition of a variable region than without the addition of a variable region.

### *In-silico* immune simulation

C-ImmSim (https://kraken.iac.rm.cnr.it/C-IMMSIM/) server was used to simulate the host immune system in response to the vaccine antigen (24). In mammals, 3 injections are given with an interval of 4 weeks apart, and the injection time steps are 1, 84, and 168, respectively (25).

## Results

### Amino acid sequence of proteins

The GenBank accession number of the Omp2b protein is AMM72579 and version AMM72579.1, with a total length of 362 amino acids. The amino acid sequence of the Omp2b protein is MNIKSLLLGSAAALVAASGAQAADAIVAPEPEAVEYVRVCDAYGAGYFYIPGTETCLRVHGYVRYDVKGGDDVYSGTDRNGWDKGARFALRVSTGSETELGTLKTFTELRFNYAANNSGVDGKYGNETSSGTVMEFAYIQLGGLRVGIDESEFHTFTGYLGDVINDDVISAGSYRTGKIAYTFTGGNGFSAVIALEQGGDNDGGYTGTTNYHIDGYMPDVVGGLKYAGGWGSIAGVVAYDSVIEEWAAKVRGDVNITDQFSVWLQGAYSSAATPDQNYGQWGGDWAVWGGLKYQATQKAAFNLQAAHDDWGKTAVTANVAYELVPGFTVTPEVSYTKFGGEWKNTVAEDNAWGGIVRFQRSF.

The GenBank accession number of the Omp31 protein is AAS84567 and version AAS84567.1, with a total length of 240 amino acids. The amino acid sequence of the Omp31 protein is MKSVILASIAAMFATSAMAADVVVSEPSAPTAAPVDTFSWTGGYIGINAGYAGGKFKHPFSSFDKEDNEQVSGSLDVTAGGFVGGVQAGYNWQLDNGVVLGAETDFQGSSVTGSISAGASGLEGKAETKVEWFGTVRARLGYTATERLMVYGTGGLAYGKVKSAFNLGDDASALHTWSDKTKAGWTLGAGAEYAINNNWTLKSEYLYTDLGKRNLVDVDNSFLESKVNFHTVRVGLNYKF.

The GenBank accession number of the CTLA-4 is P16410 and version P16410.3, with a total length of 223 amino acids. The amino acid sequence of CTLA-4 is MACLGFQRHKAQLNLATRTWPCTLLFFLLFIPVFCKAMHVAQPAVVLASSRGIASFVCEYASPGKATEVRVTVLRQADSQVTEVCAATYMMGNELTFLDDSICTGTSSGNQVNLTIQGLRAMDTGLYICKVELMYPPPYYLGIGNGTQIYVIDPEPCPDSDFLLWILAAVSSGLFFYSFLLTAVSLSKMLKKRSPLTTGVYVKMPPTEPECEKQFQPYFIPIN. Among them, the range of variable area (IgV_CTLA-4) is 39~152, its sequence is HVAQPAVVLASSRGIASFVCEYASPGKATEVRVTVLRQADSQVTEVCAATYMMGNELTFLDDSICTGTSSGNQVNLTIQGLRAMDTGLYICKVELMYPPPYYLGIGNGTQIYVI.

### Physicochemical parameters of proteins

The Omp2b protein is composed of 362 amino acids. The molecular weight is 38,676.57, the theoretical pI is 4.58, and the chemical molecular formula is C1734H2578N456O544S5. The instability index (II) is 23.54. This classifies the protein as stable. The grand average of hydrophobicity (GRAVY) is -0.200, indicating hydrophilicity.

The Omp31 protein is composed of 240 amino acids. The molecular weight is 25,323.20, the theoretical pI is 5.22, and the chemical molecular formula is C1135H1725N297O354S4. The instability index (II) is 8.61. This classifies the protein as stable. The grand average of hydrophobicity (GRAVY) is -0.091, indicating hydrophilicity.

### Signal peptide of proteins

**Figure 1:**
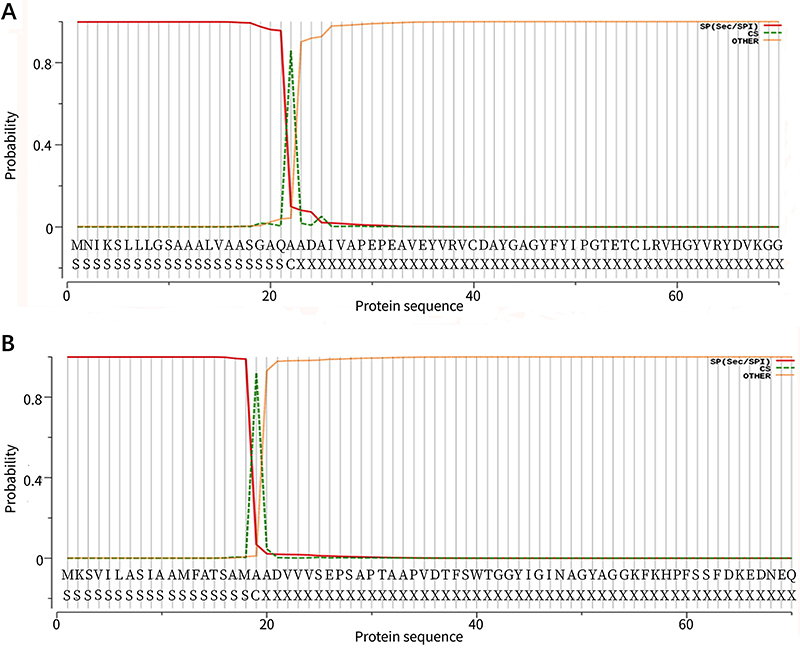
Prediction of Omp2b signal peptide (**A**) and of Omp31 signal peptide (**B**).

The probability of signal peptide of Omp2b is 99.854%, and the type of signal peptide is SP (SEC/SPI). The cleavage site is 22-23 and probability is 85.780%. The signal peptide sequence is MNIKSLLGSAAALVAASGAQA. The probability of signal peptide of Omp31 is 99.928%, and the type of signal peptide is SP (SEC/SPI). The cleavage site is 19-20 and probability is 92.130%. The signal peptide sequence is MKSVILASIAAMFATSAMA (Figure 1).

### Transmembrane domains

**Figure 2:**
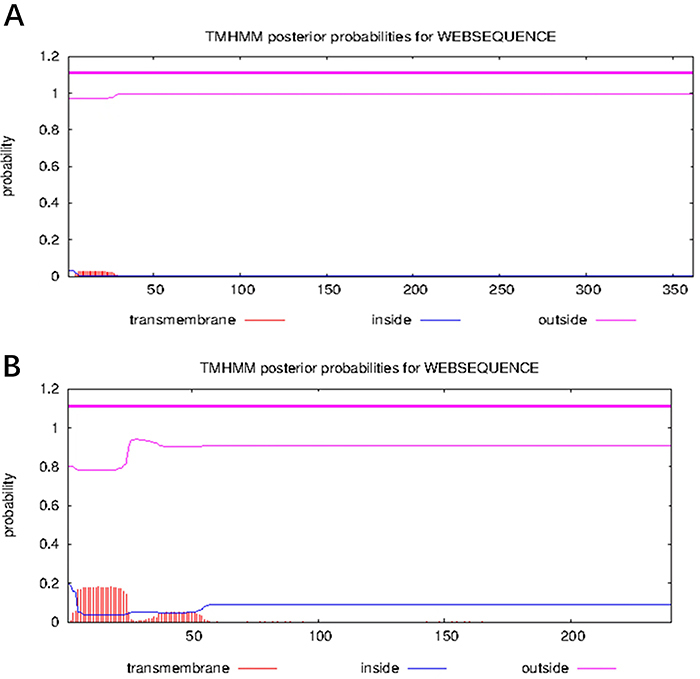
Prediction of extracellular domain. Prediction of Omp2b (**A**) and of Omp31 (**B**). As shown, Omp2b and Omp31 have no transmembrane region.

The online software TMHMM showed that both the Omp2b and Omp31 proteins did not have transmembrane domains (Figure 2).

### Analysis of T-cell and B-cell epitopes of proteins

The results of CD4+ T-cell epitopes of proteins Omp2b and Omp31 (signal peptide sequence removed) are shown in Table 1.

**Table 1:** Predicted T epitopes of Omp2b proteins and Omp31 proteins.

	Predicted T epitopes of Omp2b proteins		
Allele	Position	Sequence	Percentile rank
HLA-DRB1*07:01	199	GDNDGGYTGTTNYHI	2.40
HLA-DRB1*07:01	200	DNDGGYTGTTNYHID	2.50
HLA-DRB1*07:01	202	DGGYTGTTNYHIDGY	2.60
HLA-DRB1*07:01	203	GGYTGTTNYHIDGYM	2.60
HLA-DRB1*07:01	201	NDGGYTGTTNYHIDG	2.70
HLA-DRB1*07:01	316	TANVAYELVPGFTVT	4.40
HLA-DRB1*07:01	315	VTANVAYELVPGFTV	4.40
HLA-DRB1*07:01	317	ANVAYELVPGFTVTP	4.50
HLA-DRB1*07:01	318	NVAYELVPGFTVTPE	4.60
HLA-DRB1*07:01	319	VAYELVPGFTVTPEV	4.70
	**Predicted T epitopes of Omp31 proteins**		
**Allele**	**Position**	**Sequence**	**Percentile rank**
HLA-DRB1*07:01	158	VRARLGYTATERLMV	1.70
HLA-DRB1*07:01	160	ARLGYTATERLMVYG	2.10
HLA-DRB1*07:01	150	TKVEWFGTVRARLGY	2.10
HLA-DRB1*07:01	149	ETKVEWFGTVRARLG	2.40
HLA-DRB1*07:01	151	KVEWFGTVRARLGYT	2.50
HLA-DRB1*07:01	157	TVRARLGYTATERLM	2.50
HLA-DRB1*07:01	156	GTVRARLGYTATERL	2.70
HLA-DRB1*07:01	152	VEWFGTVRARLGYTA	3.10
HLA-DRB1*07:01	161	RLGYTATERLMVYGT	3.80

The results of B-cell epitopes of proteins Omp2b and Omp31 (signal peptide sequence removed) are shown in Table 2.

**Table 2:** Predicted B epitopes of Omp2b proteins and Omp31 proteins.

	Predicted B epitopes of Omp2b proteins		
Start	End	Sequence	Length
27	33	VAPEPEA	7
70	84	GDDVYSGTDRNGWDK	15
115	130	ANNSGVDGKYGNETSS	16
158	173	GYLGDVINDDVISAGS	16
199	216	GDNDGGYTGTTNYHIDGY	18
255	259	NITDQ	5
265	285	AATPDQNYGQWGGDW	15
293	298	YQATQK	6
308	312	DDWGK	5
339	350	GGEWKNTVAEDN	12
	**Predicted B epitopes of Omp31 proteins**		
**Start**	**End**	**Sequence**	**Length**
45	61	VVSEPSAPTAAPVDTFS	17
73	102	YAGGKFKPFSSDKEDNEQVSGSLDVTAG	30
112	118	YNWQLDN	7
128	150	FQGSSVTGSISAGASGLEGKAET	23
182	204	KVKSAFNLGDDASALHTWSDKTK	23
226	252	EYLYTDLGKRNLVDVDNSFLESKVNFH	27

### Tertiary structure prediction of the Omp2b and Omp31 proteins

**Figure 3:**
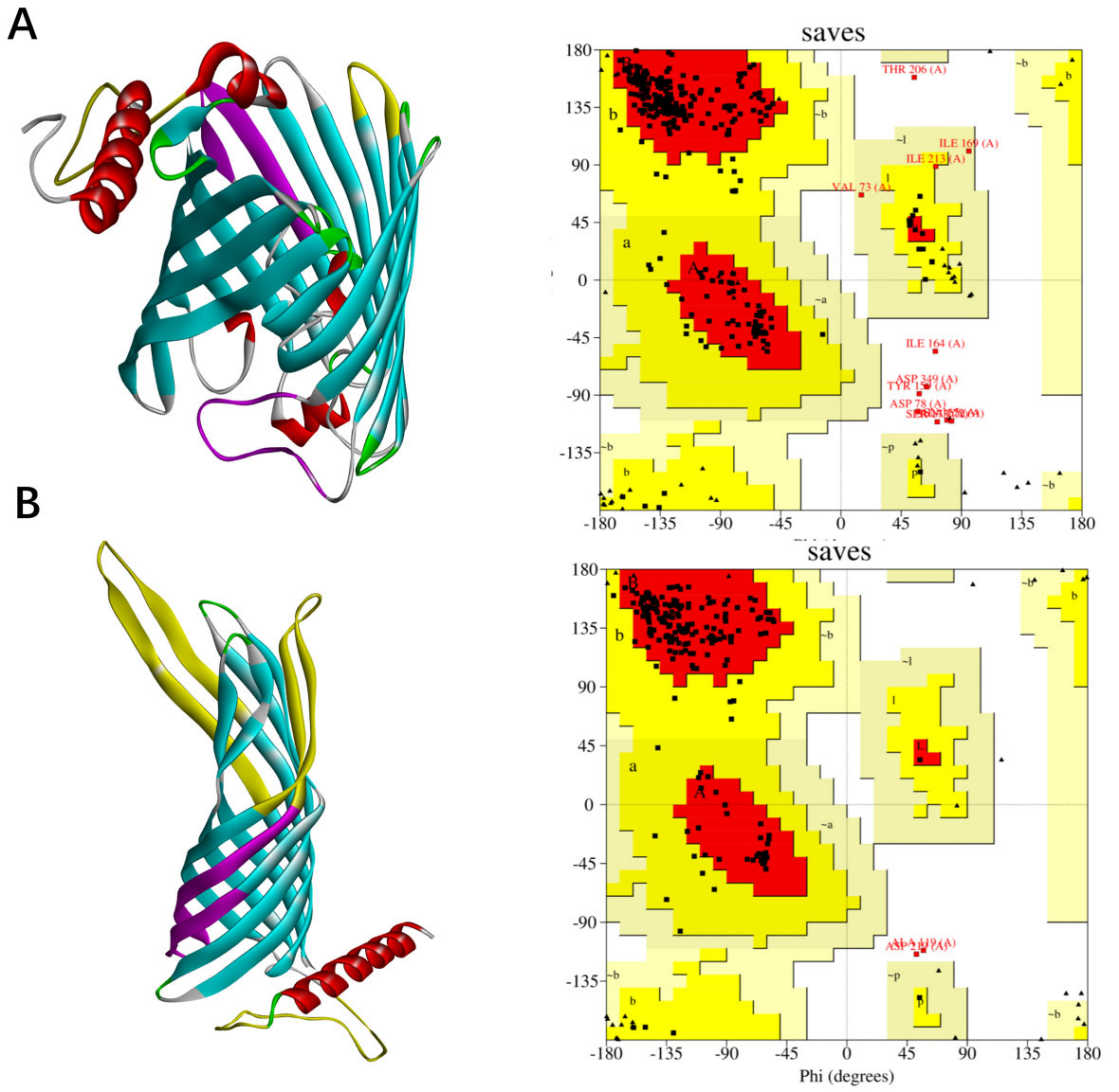
Predicted tertiary structures of Omp2b (**A**) and of Omp31 (**B**). On the left, the tertiary structure of the protein and on the right, the Ramachandran plot. The figure shows that the tertiary structures are reasonable.

All the protein molecular models were clearly viewed by the software DS. T epitope is shown in purple and B epitope is shown in yellow. We also plotted a Ramachandran plot to evaluate the rationality of structure (Figure 3).

### Analysis of epitope characteristics of the Omp2b-Omp31 fusion protein

#### Amino acid sequence of the Omp2b-Omp31 fusion protein

**Figure 4:**
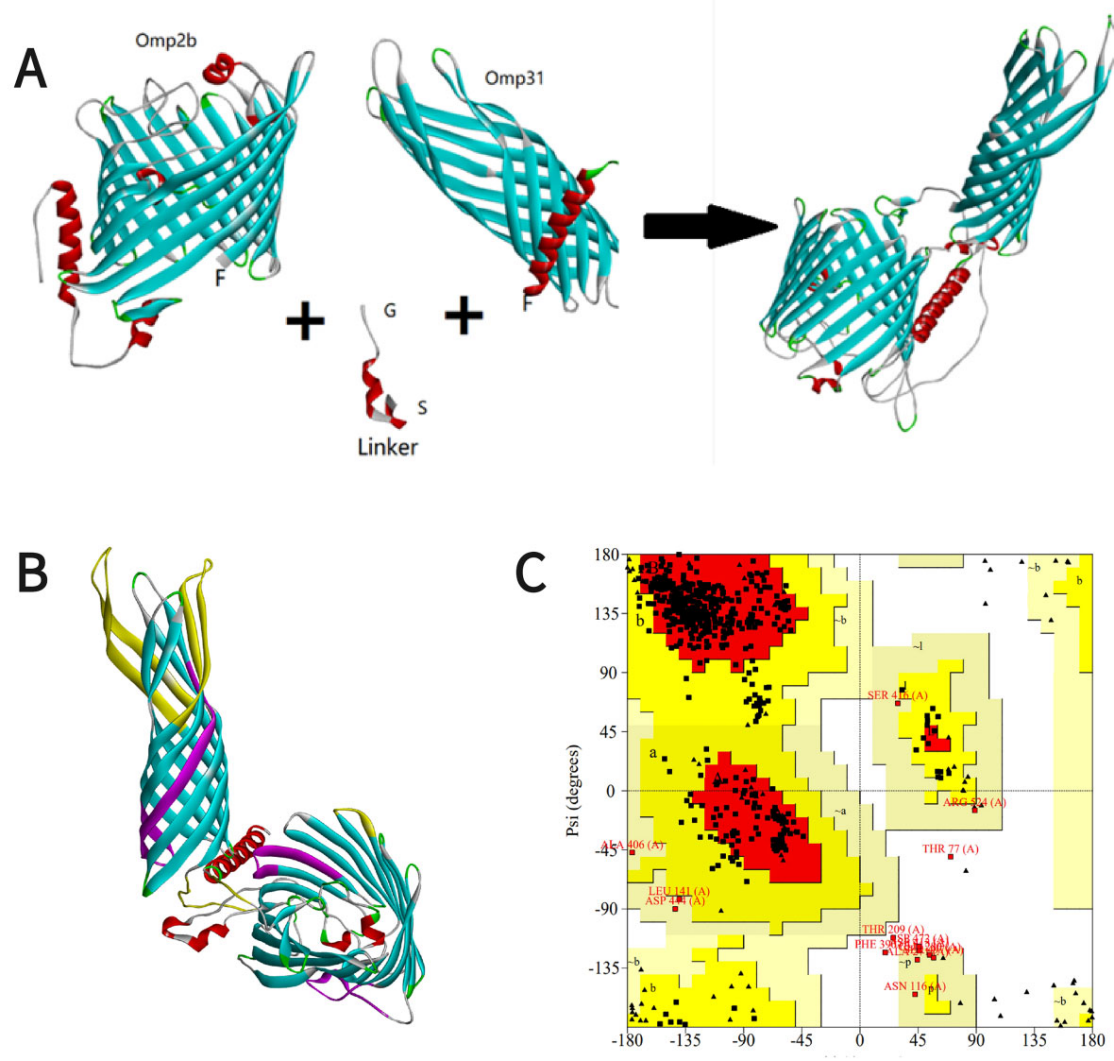
**A**, Process of fusion between Omp2b and Omp31 with linker. On the right, the arrow indicates the fusion result. **B**, Predicted tertiary structures of Omp2b-Omp31. The T epitope is shown in purple and the B epitope in yellow. **C**, Ramachandran plot. The figure shows that the tertiary structure is reasonable.

The author used the linker GGGGS (3) to connect the two proteins, named Omp2b-Omp31, as shown in Figure 4. Its sequence is MNIKSLLLGSAAALVAASGAQAADAIVAPEPEAVEYVRVCDAYGAGYFYIPGTETCLRVHGYVRYDVKGGDDVYSGTDRNGWDKGARFALRVSTGSETELGTLKTFTELRFNYAANNSGVDGKYGNETSSGTVMEFAYIQLGGLRVGIDESEFHTFTGYLGDVINDDVISAGSYRTGKIAYTFTGGNGFSAVIALEQGGDNDGGYTGTTNYHIDGYMPDVVGGLKYAGGWGSIAGVVAYDSVIEEWAAKVRGDVNITDQFSVWLQGAYSSAATPDQNYGQWGGDWAVWGGLKYQATQKAAFNLQAAHDDWGKTAVTANVAYELVPGFTVTPEVSYTKFGGEWKNTVAEDNAWGGIVRFQRSFGGGGSGGGGSGGGGSMKSVILASIAAMFATSAMAADVVVSEPSAPTAAPVDTFSWTGGYIGINAGYAGGKFKHPFSSFDKEDNEQVSGSLDVTAGGFVGGVQAGYNWQLDNGVVLGAETDFQGSSVTGSISAGASGLEGKAETKVEWFGTVRARLGYTATERLMVYGTGGLAYGKVKSAFNLGDDASALHTWSDKTKAGWTLGAGAEYAINNNWTLKSEYLYTDLGKRNLVDVDNSFLESKVNFHTVRVGLNYKF.

#### Analysis of T-cell epitope of the Omp2b-Omp31 fusion protein

The results of CD4+ T-cell epitopes of protein Omp2b-Omp31 fusion protein (signal peptide sequence removed) are shown in Table 3. By analyzing Table 3, we found that the epitopes are basically the same.

**Table 3:** Predicted T epitopes of Omp2b-Omp31 proteins and comparison with T epitopes of Omp2b and Omp31.

Omp2b			Omp2b-Omp31		
Position	Sequence	Percentile rank	Position	Sequence	Percentile rank
199	GDNDGGYTGTTNYHI	2.40	199	GDNDGGYTGTTNYHI	2.40
200	DNDGGYTGTTNYHID	2.50	200	DNDGGYTGTTNYHID	2.50
316	TANVAYELVPGFTVT	4.40	316	TANVAYELVPGFTVT	4.40
**Omp31**			**Omp2b-Omp31**		
**Position**	**Sequence**	**Percentile rank**	**Position**	**Sequence**	**Percentile rank**
159	RARLGYTATERLMVY	1.60	514	RARLGYTATERLMVY	1.60
158	VRARLGYTATERLMV	1.70	513	VRARLGYTATERLMV	1.70
160	ARLGYTATERLMVYG	2.10	515	ARLGYTATERLMVYG	2.10
150	TKVEWFGTVRARLGY	2.10	505	TKVEWFGTVRARLGY	2.10

#### Analysis of B-cell epitope of the Omp2b-Omp31 fusion protein

The results of B-cell epitopes of protein Omp2b-Omp31 fusion protein (signal peptide sequence removed) are shown in Table 4, which indicate that the epitopes are basically the same.

**Table 4:** Predicted B epitopes of Omp2b-Omp31 proteins and comparison with B epitopes of Omp2b and Omp31.

Omp2b			Omp2b-Omp31		
Position	Sequence	Percentile rank	Position	Sequence	Percentile rank
27	VAPEPEA	7	27	VAPEPEA	7
199	GDNDGGYTGTTNYHIDGY	18	199	GDNDGGYTGTTNYHIDGY	18
255	NITDQ	5	255	NITDQ	5
**Omp31**			**Omp2b-Omp31**		
**Position**	**Sequence**	**Percentile rank**	**Position**	**Sequence**	**Percentile rank**
45	VVSEPSAPTAAPVDTFS	17	397	ADVVVSEPSAPTAAPVDTFS	20
73	YAGGKFKHPFSSFDKEDNEQVSGSLDVTAG	30	429	AGGKFKHPFSSFDKEDNEQVSGSLDVTAG	29
182	KVKSAFNLGDDASALHTWSDKTK	23	539	KSAFNLGDDASALHTWSDKTK	21

#### Tertiary structure prediction of the Omp2b-Omp31 fusion protein

All the protein molecular models were viewed by software DS clearly. T epitope is shown in purple and B epitope is shown in yellow. We also plotted a Ramachandran plot to evaluate the rationality of structure (Figure 4).

### Analysis of epitope characteristics of the IgV_CTLA-4-Omp2b-Omp31 fusion protein

#### The amino acid sequence of the IgV_CTLA-4-Omp2b-Omp31 fusion protein

**Figure 5:**
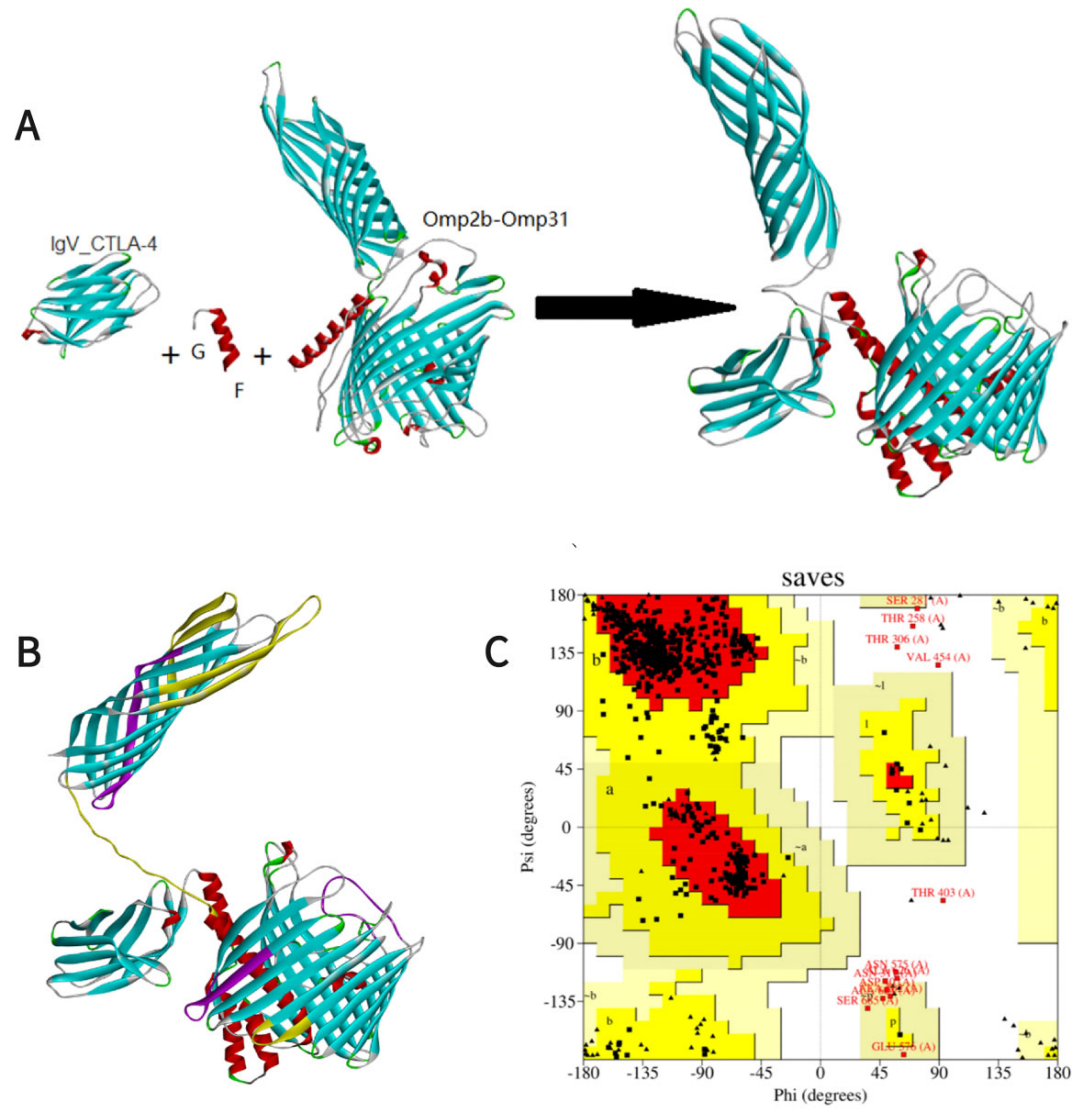
**A**, The process of fusion of Omp2b-Omp31and IgV_CTLA-4 with linker. On the right, the arrow indicates the fusion result. **B**, Predicted tertiary structures of IgV_CTLA-4-Omp2b-Omp31. The T epitope is shown in purple and the B epitope in yellow. **C**, Ramachandran plot. The figure shows that the tertiary structure is reasonable.

Through fusion, we obtained the sequence of fusion protein IgV_CTLA-4-Omp2b-Omp31 as shown in Figure 5: RFALRVSTGSETELGTLKTFTELRFNYAANNSGVDGKYGNETSSGTVMEFAYIQLGGLRVGIDESEFHTFTGYLGDVINDDVISAGSYRTGKIAYTFTGGNGFSAVIALEQGGDNDGGYTGTTNYHIDGYMPDVVGGLKYAGGWGSIAGVVAYDSVIEEWAAKVRGDVNITDQFSVWLQGAYSSAATPDQNYGQWGGDWAVWGGLKYQATQKAAFNLQAAHDDWGKTAVTANVAYELVPGFTVTPEVSYTKFGGEWKNTVAEDNAWGGIVRFQRSFGGGGSGGGGSGGGGSMKSVILASIAAMFATSAMAADVVVSEPSAPTAAPVDTFSWTGGYIGINAGYAGGKFKHPFSSFDKEDNEQVSGSLDVTAGGFVGGVQAGYNWQLDNGVVLGAETDFQGSSVTGSISAGASGLEGKAETKVEWFGTVRARLGYTATERLMVYGTGGLAYGKVKSAFNLGDDASALHTWSDKTKAGWTLGAGAEYAINNNWTLKSEYLYTDLGKRNLVDVDNSFLESKVNFHTVRVGLNYKF.

#### Analysis of T-cell epitope of the IgV_CTLA-4-Omp2b-Omp31 fusion protein

The results of CD4+ T-cell epitopes of Omp2b-Omp31 fusion protein (signal peptide sequence removed) are shown in Table 5, which indicate that the epitopes are basically the same.

**Table 5:** Predicted T epitopes of IgV_CTLA-4-Omp2b-Omp31 proteins and comparison with T epitopes of Omp2b, Omp31, and Omp2b-Omp31.

Omp2b		Omp2b-Omp31		IgV_CTLA-4-Omp2b-Omp31	
Position	Sequence	Position	Sequence	Position	Sequence
177	GDNDGGYTGTTNYHI	177	GDNDGGYTGTTNYHI	329	GDNDGGYTGTTNYHI
178	DNDGGYTGTTNYHID	178	DNDGGYTGTTNYHID	330	DNDGGYTGTTNYHID
294	TANVAYELVPGFTVT	294	TANVAYELVPGFTVT	446	TANVAYELVPGFTVT
**Omp31**		**Omp2b-Omp31**		**IgV_CTLA-4-Omp2b-Omp31**	
**Position**	**Sequence**	**Position**	**Sequence**	**Position**	**Sequence**
118	RARLGYTATERLMVY	473	RARLGYTATERLMVY	644	RARLGYTATERLMVY
117	VRARLGYTATERLMV	472	VRARLGYTATERLMV	643	VRARLGYTATERLMV
109	TKVEWFGTVRARLGY	464	TKVEWFGTVRARLGY	635	TKVEWFGTVRARLGY

#### Analysis of B-cell epitope of the IgV_CTLA-4-Omp2b-Omp31 fusion protein

The results of B-cell epitopes of IgV_CTLA-4-Omp2b-Omp31 fusion protein (signal peptide sequence removed) are shown in Table 6, which indicate that that the epitopes are basically the same.

**Table 6:** Predicted B epitopes of IgV_CTLA-4-Omp2b-Omp31 proteins and comparison with B epitopes of Omp2b, Omp31, and Omp2b-Omp31.

Omp2b		Omp2b-Omp31		IgV_CTLA-4-Omp2b-Omp31	
Position	Sequence	Position	Sequence	Position	Sequence
177	GDNDGGYTGTTNYHIDGY	177	GDNDGGYTGTTNYHIDGY	329	GDNDGGYTGTTNYHIDGY
233	NITDQ	233	NITDQ	385	NITDQ
**Omp31**		**Omp2b-Omp31**		**IgV_CTLA-4-Omp2b-Omp31**	
**Position**	**Sequence**	**Position**	**Sequence**	**Position**	**Sequence**
4	VVSEPSAPTAAPVDTFS	356	ADVVVSEPSAPTAAPVDTFS	529	VVSEPSAPTAAPVDTFS
32	YAGGKFKHPFSSFDKEDNEQVSGSLDVTAG	388	AGGKFKHPFSSFDKEDNEQVSGSLDVTAG	559	AGGKFKHPFSSFDKEDNEQVSGSLDVTAG
141	KVKSAFNLGDDASALHTWSDKTK	498	KSAFNLGDDASALHTWSDKTK	669	KSAFNLGDDASALHTWSDKTK

#### Tertiary structure prediction of the IgV_CTLA-4-Omp2b-Omp31 fusion protein

With the same method as above, we obtained the tertiary structure of the IgV_CTLA-4-Omp2b-Omp31 fusion protein and the Ramachandran plot (Figure 5). By comparing the tertiary structure of the above proteins, it was not difficult to see that their T/B epitopes did not change, showing that the fused protein is not affected by the linker.

### Molecular docking

**Figure 6:**
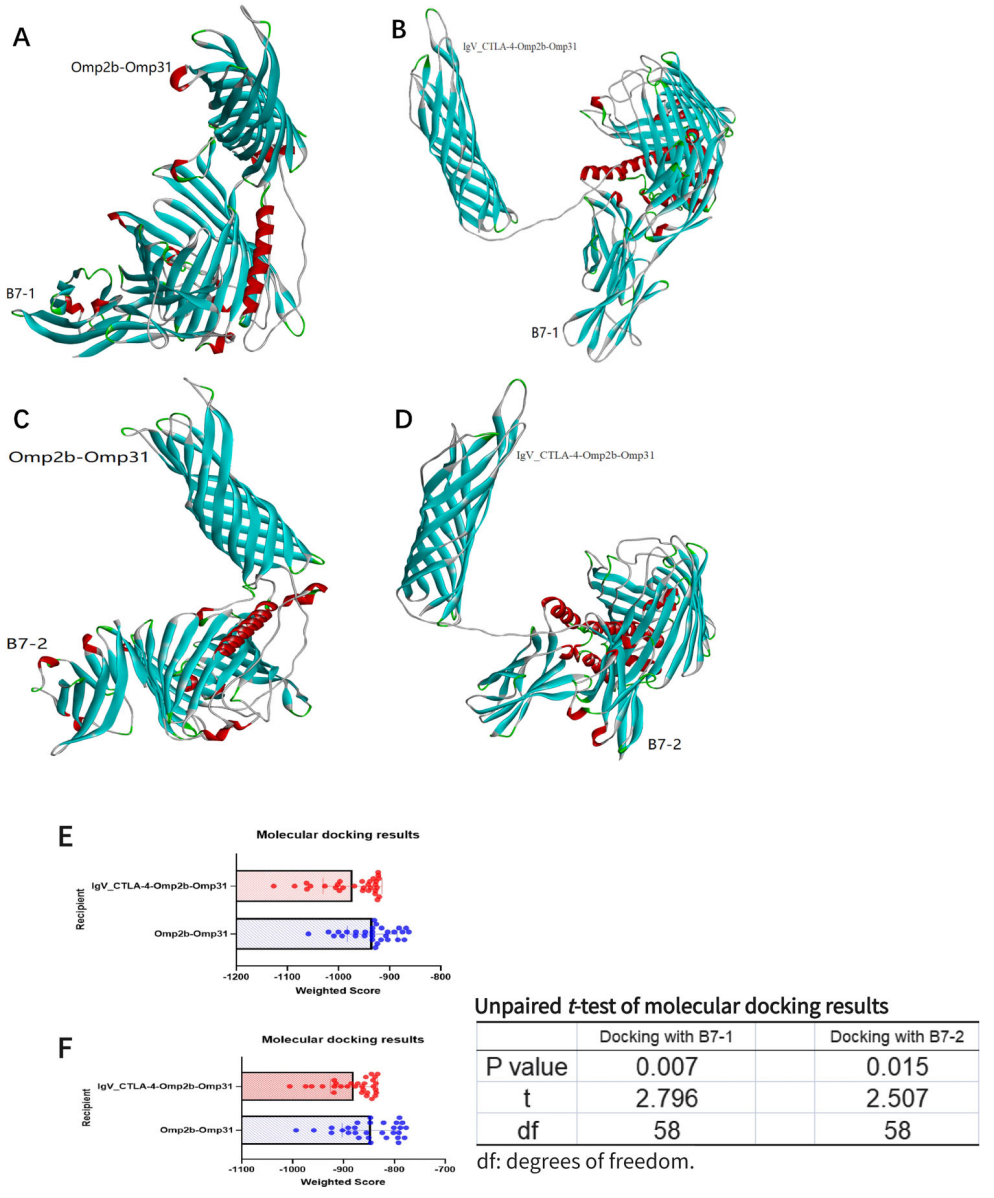
**A**, Docking of B7-1 and Omp2b-Omp31; **B**, Docking of B7-1 and IgV_CTLA-4-Omp2b-Omp31; **C**, Docking of B7-2 and Omp2b-Omp31; **D**, Docking of B7-2 and IgV_CTLA-4-Omp2b-Omp31; **E**, Statistical diagram of B7-1 docking with Omp2b-Omp31 and IgV_CTLA-4-Omp2b-Omp31, which indicates that the docking effect of IgV_CTLA-4-Omp2b-Omp31 is stronger than that of Omp2b-Omp31; **F**, Statistical diagram of B7-2 docking with Omp2b-Omp31 and IgV_CTLA-4-Omp2b-Omp31, which indicates that the docking effect of IgV_CTLA-4-Omp2b-Omp31 is stronger than that of Omp2b-Omp31.

B7-1 and B7-2 were used for molecular docking with Omp2b-Omp31 and IgV_CTLA-4-Omp2b-Omp31 as shown in Figure 6.

The score of B7-1 and B7-2 docking with the two fusion proteins was tested by two independent samples *t*-test, as shown in Figure 6.

We concluded that whether docking with B7-1 or B7-2, the affinity of the fusion protein with variable region was indeed different from that without the variable region, and the affinity of the former was stronger than that of the latter (P<0.05). Therefore, IgV_CTLA-4-Omp2b-Omp31 induced stronger immune responses than Omp2b-Omp31.

### *In-silico* immune simulation

**Figure 7:**
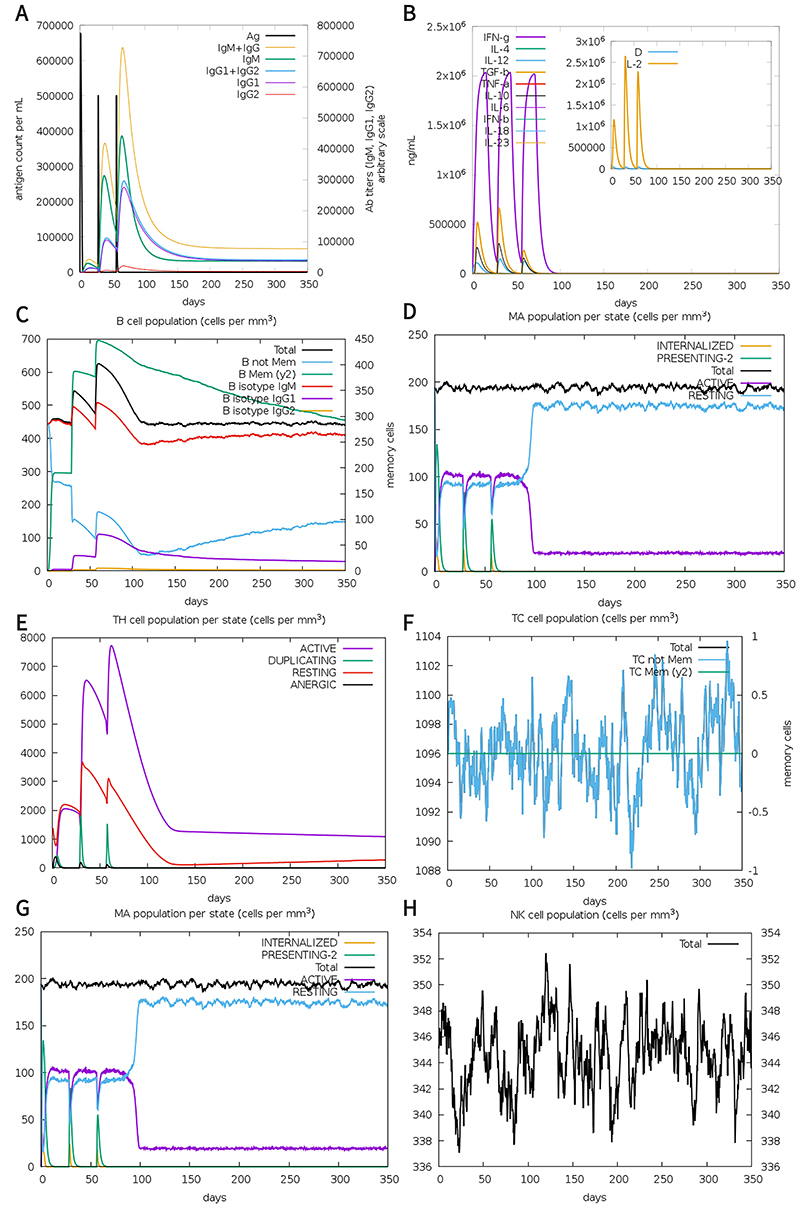
The results of immune simulation. **A**, Level of antigen and antibody after vaccination; **B**, Cytokine level; **C**, B cell level; **D**, Dendritic cell (DC) level; **E**, TH cell level; **F**, Killer T cell (TC) level; **G**, Macrophage (MA) level; **H**, Natural killer (NK) cell level.

The *in silico* immune simulator was used to evaluate the immune response caused by the actual vaccine injected into the human body. Overall, the primary immune response mainly causes the increase of IgM and IgG, and the secondary immune response mainly causes the increase of IgG1, IgG1+IgG2, and B cells. In addition, vaccines can also cause intense cytokine reactions, the most important of which is elevated levels of interferon, IL-23, IL-10, and IL-12. Most importantly, the T cell epitope in the vaccine has immunogenicity, which is consistent with the previous prediction. In addition, after each vaccine injection, the number of macrophages increased, and most natural killer cells remained at a high level during the whole immune process, as shown in Figure 7.

## Discussion

In many countries, *Brucella* infection is still a major threat to livestock and humans (26). Since vaccines remain the first choice for brucellosis prevention, the development of an effective vaccine against brucellosis is particularly important. In recent years, with the rapid development of molecular biology, nucleic acid and protein bioinformatics technology has become simple and accurate (27). Using bioinformatics methods to design novel fusion protein vaccines composed of multiple single proteins has become a new way to induce protective immune responses (28). At present, the development of brucellosis vaccine has changed from a univalent vaccine to a multivalent compound vaccine (29). According to previous studies, Omp2b and Omp31 proteins have good antigenicity as candidate molecules for brucellosis vaccine. Therefore, we selected these two proteins to design a fusion protein vaccine. Moreover, the research showed that it has excellent effectiveness.

In a previous study, Gan et al. (30) constructed the fusion protein with linker and proved the feasibility of this method through experiments. Therefore, bioinformatics methods were used to construct the fusion protein Omp2b-Omp31. The proteins Omp2b, Omp31, Omp2b-Omp31, and IgV_CTLA-4-Omp2b-Omp31 were analyzed by bioinformatics method. First of all, we defined the amino acid sequences of Omp2b and Omp31 by NCBI. Through their sequences, we predicted the physicochemical parameters of protein Omp2b and Omp31. The analysis found that Omp2b and Omp31 are stable and hydrophilic proteins, which are favorable to the construction of vaccine. Then, we fused the two proteins with linker (GGGGS)3 to enhance the vaccine immunogenicity. Glycine G and serine S are usually selected as the constituent amino acids of linker sequence because they are the smallest of all amino acids and have no chiral carbon, so they are the most flexible, and will not affect the conformation and function of the two proteins when placed between fusion proteins (31). Furthermore, we predicted the T/B epitopes of Omp2b, Omp31, and Omp2b-Omp31, and it was found that the fused protein T/B epitope was almost identical to the previous epitopes of Omp2b and Omp31. In addition, we presented those proteins with a tertiary structure model by DS and determined the rationality of their tertiary structures by drawing Ramachandran diagrams. This method not only maintains the T/B epitope, but also may enhance the immunogenicity of the vaccine, enhancing the immune response of the vaccine.

In order to increase the affinity between protein and APCs and increase its antigen presentation ability, CTLA-4 was fused with the protein in this study. Previous studies demonstrated that fusing CTLA-4 with antigens significantly improves specific immune responses (32). Based on the efficient binding between CTLA-4 variable region and B7 molecule on antigen-presenting cells, CTLA-4 variable region is fused with Omp2b-Omp31 antigen, so that the antigen can accurately bind to APCs and produce a strong immune response (33). The T/B epitope of the IgV_CTLA-4-Omp2b-Omp31 fusion protein was predicted and compared with other proteins, as mentioned above. The results showed that the sequence and the number of Omp2b-Omp31 T/B epitope did not change after IgV_CTLA-4 was linked to the N-terminal of Omp2b-Omp31. Using the same method, we constructed the tertiary structure of IgV_CTLA-4-Omp2b-Omp31, marked the T/B epitope by DS, and evaluated its rationality with the Ramachandran diagram. It was observed that the addition of the linker sequence made the adjacent proteins before and after it fold normally and did not affect their respective functions. B7-1 and B7-2 were docked with Omp2b-Omp31 and IgV_CTLA-4-Omp2b-Omp31. The results showed that there were differences between the two scores and that the docking effect of IgV_CTLA-4-Omp2b-Omp31 was better than that of Omp2b-Omp31.

In summary, we learned that the fusion of the two antigens and the addition of IgV_CTLA-4 as an adjuvant not only had no effect on the epitope of the antigen itself, but also improved the antigenicity and immunogenicity. At this point, we can obtain an excellent fusion vaccine. The results of this study can serve as a reference for the design of an effective brucellosis vaccine.
